# Iron Oxide Nanocrystals for Magnetic Hyperthermia Applications

**DOI:** 10.3390/nano2020134

**Published:** 2012-05-07

**Authors:** Leisha M. Armijo, Yekaterina I. Brandt, Dimple Mathew, Surabhi Yadav, Salomon Maestas, Antonio C. Rivera, Nathaniel C. Cook, Nathan J. Withers, Gennady A. Smolyakov, Natalie Adolphi, Todd C. Monson, Dale L. Huber, Hugh D. C. Smyth, Marek Osiński

**Affiliations:** 1 Center for High Technology Materials, 1313 Goddard SE, University of New Mexico, Albuquerque, NM 87106, USA; Email: leishyne@unm.edu (L.M.A.); ybrandt@salud.unm.edu (Y.I.B.); dmathew@chtm.unm.edu (D.M.); surabhi.iitd@gmail.com (S.Y.); salomon@unm.edu (S.M.); arivera@chtm.unm.edu (A.C.R.); ncook93@unm.edu (N.C.C.); nwithers@chtm.unm.edu (N.J.W.); gen@chtm.unm.edu (G.A.S.); 2 Department of Biochemistry and Molecular Biology, Health Sciences Center, University of New Mexico, Albuquerque, NM 87131, USA; Email: nadolphi@salud.unm.edu; 3 Sandia National Laboratories, Nanomaterials Sciences, P.O. Box 5800, MS 1415, Albuquerque, NM 87185, USA; Email: tmonson@sandia.gov; 4 Center for Integrated Nanotechnologies, Sandia National Laboratories, 1000 Eubank SE, Albuquerque, NM 87123, USA; Email: dlhuber@sandia.gov; 5 College of Pharmacy, University of Texas at Austin, Austin, TX 78712, USA; Email: hsmyth@mail.utexas.edu

**Keywords:** iron oxide nanocrystals, hyperthermia, thermotherapy, ferrofluid

## Abstract

Magnetic nanocrystals have been investigated extensively in the past several years for several potential applications, such as information technology, MRI contrast agents, and for drug conjugation and delivery. A specific property of interest in biomedicine is magnetic hyperthermia—an increase in temperature resulting from the thermal energy released by magnetic nanocrystals in an external alternating magnetic field. Iron oxide nanocrystals of various sizes and morphologies were synthesized and tested for specific losses (heating power) using frequencies of 111.1 kHz and 629.2 kHz, and corresponding magnetic field strengths of 9 and 25 mT. Polymorphous nanocrystals as well as spherical nanocrystals and nanowires in paramagnetic to ferromagnetic size range exhibited good heating power. A remarkable 30 °C temperature increase was observed in a nanowire sample at 111 kHz and magnetic field of 25 mT (19.6 kA/m), which is very close to the typical values of 100 kHz and 20 mT used in medical treatments.

## 1. Introduction

Iron oxide nanocrystals are ideal materials for biomedical applications, due to their well-established biocompatibility [1] and inherent multifunctionality [2]. For example, iron oxide nanoparticles have received FDA approval for use in humans in magnetic resonance imaging (MRI) as contrast agents [3,4]. They have also been tested in Phase 1 and Phase 2 trials for hyperthermic antitumor therapy in Germany, and seem to exhibit no systemic toxicity [5,6]. This application exploits the ability of magnetic nanoparticles to release thermal energy upon placement in an external oscillating magnetic field (magnetic hyperthermia) [7,8]. In addition, magnetic nanoparticles hold potential as efficient drug carriers [9,10], since they may be guided by the magnetic field toward a specific area of interest, thereby reducing the required dose and eliminating systemic side effects.

In the frequency range suitable for human patient treatment, three potential mechanisms are implicated in heating: Néel relaxation, Brownian motion relaxation, and hysteresis losses in the ferro-ferrimagnetic size range. This phenomenon is exploited in the application of hyperthermic tumor destruction, or thermotherapy. Furthermore, thermal energy from magnetic hyperthermia can potentially be used to trigger drug release from the nanocrystals, once the magnetic nanocrystals have reached the area of interest.

In this paper, we report on the synthesis and characterization of iron oxide nanocrystals capped with polyethylene glycol (PEG) to enhance solubility of nanocrystals in water and reduce their oxidation and aggregation. Iron oxide nanocrystals of various sizes and morphologies were synthesized and characterized by transmission electron microscopy (TEM), X-ray diffraction (XRD), and energy dispersive X-ray spectroscopy (EDS), and tested for magnetic hyperthermia using the Magnetherm system manufactured by NanoTherics, Ltd.

## 2. Results and Discussion

### 2.1. Synthesis of Colloidal Nanocrystals

Iron oxide nanocrystals were synthesized in a high boiling point solvent consisting of inert hydrocarbons and capped with PEG. For the synthesis we used a modified procedure published elsewhere [11]. Our modifications to it yielded nanocrystals of various sizes and morphologies. Polymorphous nanocrystals as well as spherical nanocrystals and nanowires were obtained by varying the temperature of the synthesis through the use of an appropriate solvent. For hyperthermia experiments, nanocrystals in the superparamagnetic to ferromagnetic size range of ~20 nm were chosen.

### 2.2. Structural Characterization

**Figure 1:**
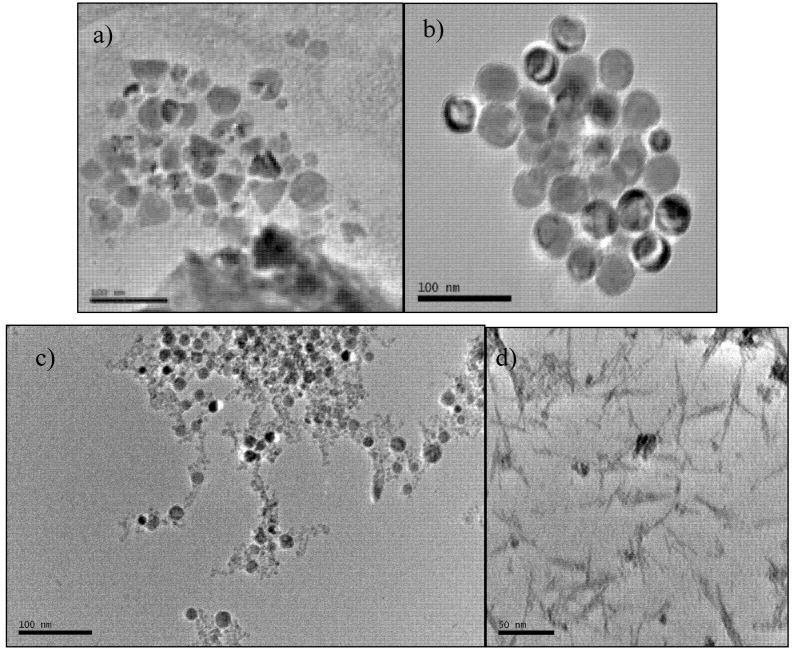
Transmission electron microscopy (TEM) images of Fe_3_O_4_ nanocrystals prior to polyethylene glycol (PEG) capping: (**a**) polymorphous nanocrystals, scale bar 100 nm; (**b**) monodisperse spheres formed from polymorphous nanocrystals, scale bar 100 nm; (**c**) monodispere spheres ~22 nm in diameter, scale bar 100 nm; (**d**) nanowires, scale bar 50 nm.

**Figure 2:**
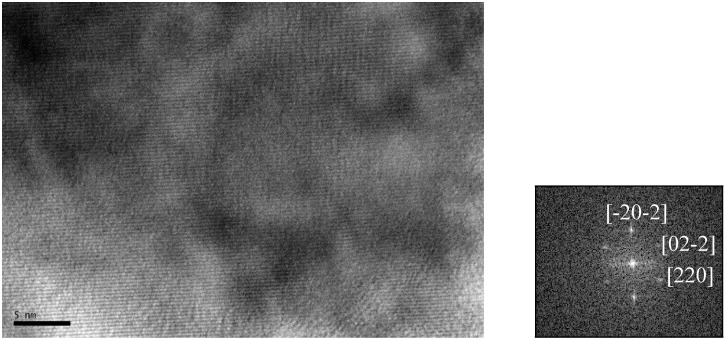
High-resolution TEM image and its Fast Fourier transform for the monodisperse spheres shown in Figure 1b above.

The TEM images in Figure 1, Figure 2 show the various morphologies and sizes of Fe_3_O_4_ nanocrystals we were able to obtain prior to PEG capping. Polymorphous nanocrystals shown in Figure 1a were obtained with eicosane solvent (342.7 °C boiling point). Monodisperse spheres with a diameter of ~ 30 nm in Figure 1b formed from polymorphous nanocrystals shown in Figure 1a) when the reaction mixture was allowed to cool for 30 min before being refluxed again. Spheres of ~22 nm in diameter (Figure 1c and nanowires 55 × 2 nm (Figure 1d) were made in *n*-docosane (boiling point 370 °C) and *n*-dodecane (boiling point 216.2 °C), respectively. We performed high-resolution TEM to confirm high crystallinity of the nanocrystals. Image in Figure 2 represents fringes observed for the monodisperse spheres from Figure 1b. 

**Figure 3:**
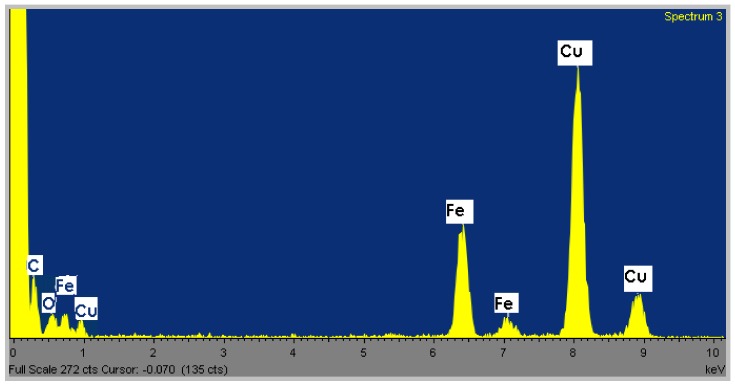
Energy dispersive X-ray spectroscopy (EDS) analysis of the monodisperse spheres from Figure 1b.

Elemental composition of the Fe_3_O_4 _nanocrystals was determined by EDS analysis. Iron and oxygen are present in monodisperse spheres from Figure 1b, confirming their elemental composition (Figure 3). The carbon and copper peaks are due to the carbon-coated copper grid.

**Figure 4:**
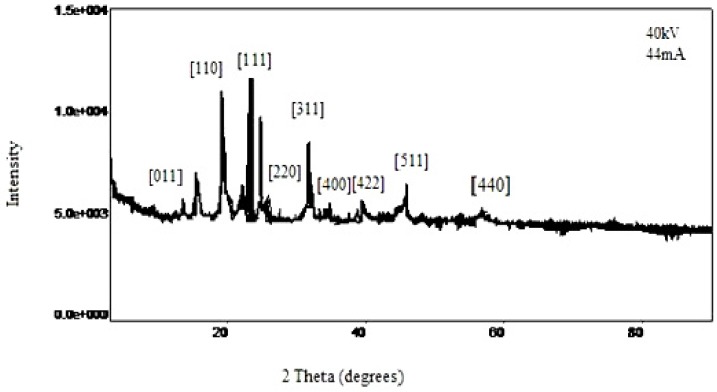
X-ray diffraction (XRD) data for iron oxide polymorphous nanocrystals.

The XRD data for iron oxide polymorphous nanocrystals (Figure 4) suggests that the composition of the nanocrystals is 70% Fe_3_O_4_ with space group Fd3m {F41/d 3 2/m}. There are peaks distinctive of *α*-Fe_2_O_3,_ which are likely the result of surface oxidation during the analysis. The remaining portions of the crystal appear to be composed of a yet unidentified phase of Fe_2_O_3_ in addition to wüstite phases, as we would expect for diiron (III) oxide, although with space groups and *a* values being similar, the oxidation state is difficult to determine with absolute certainty.

### 2.3. Magnetic Characterization

A typical feature in magnetic nanocrystals is their irreversible ferromagnetic behavior below the blocking temperature *T*_B_ and reversible magnetization above it, caused by superparamagnetic behavior of the nanocrystals. The blocking temperature can be found experimentally by measuring magnetization under field-cooling (FC) and zero-field cooling (ZFC) conditions. Below *T*_B_, the Néel relaxation time τ_N_ is larger than the measurement time τ_m_ (typically 100 s), and magnetization depends strongly on the field history. Above *T*_B_, magnetization is strongly affected by thermal fluctuations (τ_m_ > τ_N_), making FC and ZFC curves coincide. In other words, for a given measurement time τ_m_, hysteretic behavior observed below *T*_B_ would not be observed above *T*_B_.

**Figure 5:**
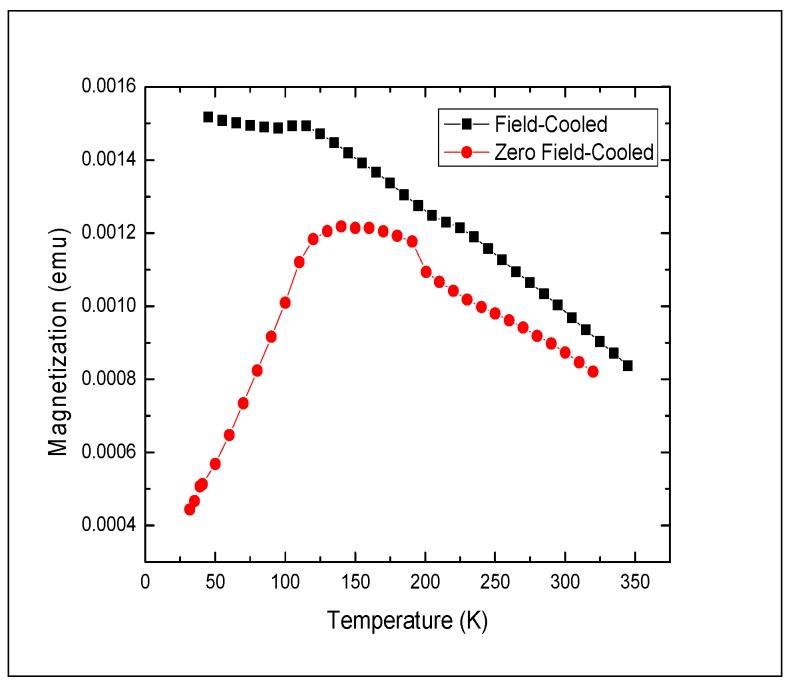
Zero-field cooled (circle symbols) and field cooled (square symbols) magnetization *vs*. temperature for polymorphous Fe_3_O_4_ nanocrystals. Magnetization measured with a dc field of 100 Oe. τ_m_ = 100 s.

We measured temperature dependence of magnetization for the Fe_3_O_4_ nanocrystal samples under ZFC and FC conditions. The dc (τ_m_ = 100 s) magnetization of the ferrofluid samples was measured with a dc field of 100 Oe in the temperature range between 9 K and 350 K.

In the entire temperature range up to 350 K, the Fe_3_O_4_ nanocrystal samples demonstrated strong ferromagnetic behavior as evidenced by the gap between the ZFC and FC curves persisting even at 350 K (Figure 5). From the ZFC curve, we can loosely estimate *T*_B_ to be ~175 K, but even above that temperature equilibrium magnetization of the nanocrystal sample was not reached, and superparamagnetic behavior of the nanocrystals was not observed.

**Figure 6:**
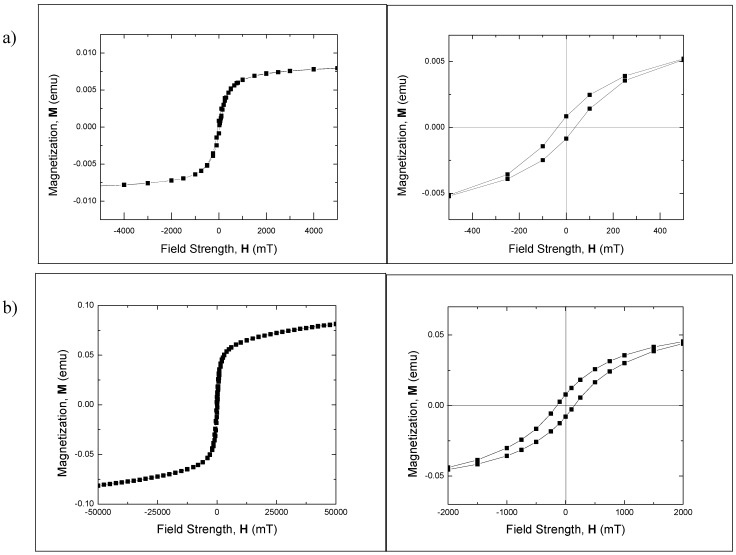
Hysteresis loop for (**a**) Fe_3_O_4_polymorphous nanocrystals and (**b**) ~22 nm Fe_3_O_4_ nanospheres. τ_m_ = 100 s. **Left**: full sweep of magnetic field measured at 293 K showing saturation. **Right**: enlarged loop measured at 293K at low field.

Strong ferromagnetic behavior of the Fe_3_O_4_ nanocrystal samples was confirmed in magnetic hysteresis measurements. Consistent with the results of dc magnetization measurements, magnetic hysteresis measurements at 293 K performed on Fe_3_O_4_ polymorphous nanocrystals (Figure 6a) find large coercivity ~37 mT (~29 kA/m) at 100 s measurement time. Even larger coercivity of ~119 mT (~94.7 kA/m) was measured for ~22 nm Fe_3_O_4_ nanospheres.

### 2.4. Magnetic Hyperthermia Experiments

Magnetic hyperthermia for the Fe_3_O_4_ nanocrystal samples was tested using a NanoTherics, Ltd. Magnetherm that operates at frequencies between 100 and 1000 kHz. Samples were prepared as described in the Experimental Section and dispersed in deionized water. All concentrations were 30 mg/mL and sample volumes were 5 mL. The nanocrystals compared in the following graphs were 22 nm spheres, polymorphous nanocrystals, and 55 × 2 nm wires. Heating of the nanocrystals was tested at frequencies of 111.1 kHz and 629.2 kHz. Data acquisition for hyperthermia was started at ambient temperature. Temperature increase was measured using an optical thermometer with an accuracy of ±0.5 °C.

**Figure 7:**
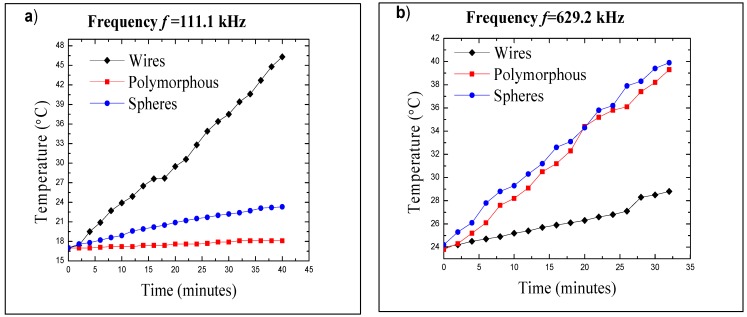
Hyperthermia data comparing the frequency dependence for different nanocrystal morphologies (22 nm spheres, polymorphous nanocrystals, and 55×2 nm wires). Taken at frequency of (**a**) 111.1 kHz (magnetic field 25 mT) and (**b**) of 629.2 kHz (magnetic field 9 mT).

Figure 7a shows the heating of nanocrystals of various morphologies (spheres, polymorphous nanocrystals, and wires) at a frequency of 111.1 kHz (magnetic field of 25 mT). Figure 7b shows the heating of the same nanocrystals at a frequency of 629.2 kHz (magnetic field of 9 mT). The spherical and polymorphous particles follow a similar trend consistent with their similar morphology and particle volume. Although they do heat at the frequency of 111.1 kHz, the observed heating was relatively low. Interestingly, the total increase in temperature after 40 min was 6 °C for spheres, whereas, it was only 1 °C for polymorphous nanocrystals. When the frequency of the oscillating magnetic field was increased to 629.2 kHz, the spheres and polymorphous nanocrystals showed increased heating. But, unlike the data obtained at 111.1 kHz, the total temperature increase was similar for the polymorphous nanocrystals and nanocrystals of spherical shape: 16 °C for spheres and 15 °C for polymorphous nanocrystals. As for the nanowires, the observed trend was just the opposite. The total temperature increase at 111.1 kHz after 40 min was remarkable 30 °C and, notably, saturation of the temperature was not reached in this timeframe. At 629.2 kHz, however, the increase of temperature generated by the wires was much less than the one obtained by spheres and polymorphous nanocrystals, representing the total temperature increase of 4 °C.

It has been shown that the transition from ferromagnetic to superparamagnetic behavior is associated with the change of the loss mechanism and, accordingly, of the heating effect of magnetic nanocrystals in hyperthermia experiments [12]. Hysteresis losses dominate in ferromagnetic nanocrystals, whereas heat production in superparamagnetic ones is due to relaxation losses. Since the blocking temperature *T*_B_ explicitly depends on the measurement time τ_m_ (inversely proportional to the frequency of the oscillating magnetic field), superparamagnetic nanocrystals, as measured in dc magnetization experiments, become ferromagnetic at sufficiently high frequencies > 1/τ_N_ (or > 1/τ, where τ = τ_Ν_ τ_Β_/(τ_Ν_ + τ_Β_), if both Néel and Brownian relaxation mechanism are present) and generate heat due to hysteresis losses. With the Fe_3_O_4_ nanocrystal samples demonstrating strong ferromagnetic behavior in dc magnetization and hysteresis measurements, hysteresis losses are expected to be the main mechanism of heating in the operating frequency range of our hyperthermia experiments. We estimated the Néel relaxation time τ_N_ at room temperature for the Fe_3_O_4_ polymorphous nanocrystals (Figure 5) as follows: τ_N_ = τ_0_exp(*E*_b_/*k*_B_*T*), where *E*_b_ is the magnetic anisotropy energy barrier, *k*_B_ is the Boltzmann constant, and τ_0_ = 10^−10^ s is the attempt time. *E*_b_ is related to the blocking temperature *T*_B_ as *E*_b_ = *k*_B_*T*_B_ ln(τ_m_/τ_0_) = 27.6 *k*_B_*T*_B_, and we arrive at τ_N_ = τ_0_exp(27.6 *T*_B_/*T*) for the Néel relaxation time. At *T* = 300 K, τ_N_ ≈ 0.001 s. At the frequencies of interest ω >> 1/τ_N_, that is far from the relaxation resonance, the Néel relaxation losses saturate at a level that is negligible for large enough τ_N_ [12]. Therefore, we will interpret our results based on the mechanism of hysteresis losses prevailing.

When hysteresis losses are the main heating factor, heating power is proportional to the area of the hysteresis loop and to the frequency of the applied magnetic field. Approximately 6-fold increase in the heating power is expected when the frequency is changed from 111.1 kHz to 629.2 kHz. The observed increase in heating power from the Fe_3_O_4_ polymorphous nanocrystals and nanospheres is not that large. We note, however, that both the frequency and amplitude of the magnetic field were changed in our experiments, and the magnetic field strength was reduced from 25 mT at 111.1 kHz to 9 mT at 629.2 kHz, which can explain the heating power increase not being proportional to the frequency for the Fe_3_O_4_ polymorphous nanocrystals and nanospheres. The higher temperature increase of 6 °C for the spheres compared to 1 °C for the polymorphous nanocrystals at 111.1 kHz can be explained by a significantly larger area of their hysteresis. However, the difference in the specific heat production between the spherical and polymorphous nanocrystals at 629.2 kHz is not that pronounced.

We consider hyperthermia experiments with nanowires separately, as their morphology differs dramatically from that of polymorphous nanocrystals and nanospheres, and may be the decisive factor. Fine magnetite particles of needle shape with high aspect ratio have been investigated previously by Hergt *et al*. [12]. High potential for hyperthermia was noted there for such particles that possess very high shape anisotropy, and hence high-energy barrier for remagnetization, resulting in a wide hysteresis and high hysteresis losses. It was concluded, however, that strong magnetic fields, very often unacceptable for human patients, are required to fully utilize their potential. Very strong nonlinear dependence of the hysteresis loss on the strength of the applied magnetic field was reported. Comparison was made among particles of different shape, and it was found that needles were by far superior when applied magnetic field exceeded ~35 kA/m, while below that value the magnetic field was not strong enough to open the hysteresis loop in needles, and their hysteresis losses were by several orders of magnitude lower compared to particles of other shapes with low aspect ratio. We expect similar effects to be observed in nanowires that are characterized with even higher aspect ratios of their shape. We believe that our results for hyperthermia in nanowires can be explained by similar superlinear dependence of their hysteresis loss on the magnetic field strength, with that superlinear dependence being much stronger than mere proportionality of the heating power to the frequency of the applied magnetic field.

We note that the remarkable 30 °C temperature increase was observed in nanowires sample at 111 kHz and magnetic field of 25 mT (19.6 kA/m), which is very close to the typical values used in medical treatments −100 kHz and 20 mT [13,14].

In our analysis, we have not considered one more possible mechanism of heat generation during hyperthermia, namely, Brownian relaxation that can be significant in particles of that size. Additional experiments are needed to clarify contribution of Brownian relaxation to the observed heat production in hyperthermia experiments. 

## 3. Experimental Section

FeCl_3_·6H_2_O (97%) was purchased from Sigma-Aldrich, *n-*docosane (99%) and *n-*eicosane (99%) were purchased from Alfa Aesar, *n-*dodecane (>99%) was purchased from Fischer Scientific, sodium oleate (>97%) was purchased from Tokyo Chemical Industry Co., PEG 3350 powder was purchased from Fisher Chemical. All chemicals were used as received, without purification.

For structural characterization, TEM/EDS samples were prepared by placing a drop of the colloidal solution onto a 200-mesh carbon-coated copper grid. The solvent was allowed to evaporate away, thus fixing the sample on the grid. The JEOL-2010F transmission electron microscope was equipped with an OXFORD Link ISIS energy dispersive spectroscopy (EDS) apparatus, which determined elemental composition. The electron beam was focused on a single nanocrystal and the characteristic X-ray peaks specific to each element were identified using the OXFORD Link ISIS software. The iron oxide phase and crystal structure was determined using a Rigaku Smartlab^®^ X-Ray Diffractometer (XRD) with a Cu K_α_ source (0.154 nm). 

The dc (τ_m_ = 100 s) magnetization of the ferrofluid samples was measured with a dc field of 100 Oe in the temperature range between 9 K and 350 K using a Quantum Design magnetic property measurement system (MPMS) superconducting quantum interference device (SQUID) magnetometer.

### 3.1. Synthesis of Iron Oleate Precursor Complex

**Figure 8:**
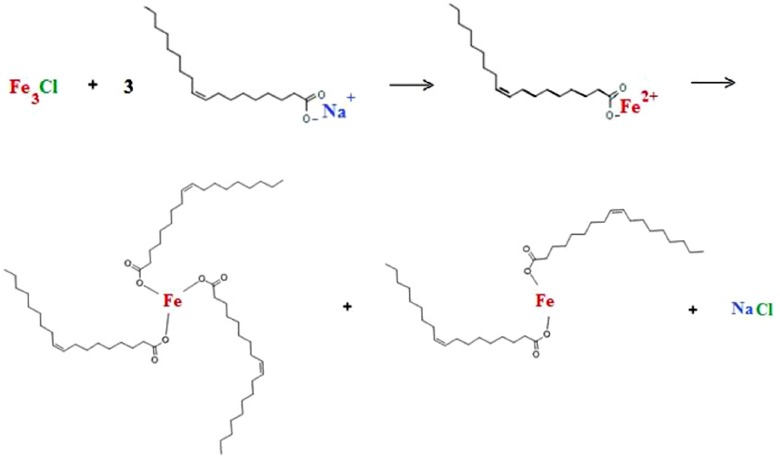
Formation of iron oleate complex from sodium oleate.

The procedure consisted of two steps: synthesis of the iron oleate precursor complex and synthesis of the iron oxide nanocrystals. The precursor was iron oleate, (iron(II,III) [(9Z)-9-octadecenoate]_n_) where n is the coordination number of iron, and could form a monomer, dimer, or trimer [15,16], produced in our laboratory using a modified procedure of Bronstein *et al.* [15]. The formation of the complex was verified with UV-Vis-NIR spectroscopy. The iron oleate complex was formed from the combination of sodium oleate salt (sodium (9Z)-9-octadecenoate) and iron(III)chloride hexahydrate (FeCl_3_·6H_2_O). In a standard reaction, 6.75 g of FeCl_3_·6H_2_O was combined with 25 mL of deionized water and vacuum-filtered through 0.22 μm filter paper. The mixture was then combined with 24.35 g of sodium oleate in a three-neck round bottom flask. 150 mL of a stock solution, consisting of a 2:4:6 mixture of deionized water, ethanol, and hexane, was added to the flask. Under argon flow, the mixture was vented and filled for three one-minute intervals, in order to remove all oxygen from the reaction flask. The solution was the slowly (5 °C/min) heated to 50 °C under vigorous stirring. Once the solid sodium oleate salt had completely melted and the reflux had begun (around 50–60 °C), the temperature was further increased (3 °C/min) to 70 °C and the flask was kept at this temperature for four hours, ensuring that the total reflux time was 4 hours. The mixture was then cooled to 60 °C and washed three times with a 1:1 mixture of hexane and deionized water in a separatory flask. The organic layer was placed in a rotary evaporator (Rotovap) with the water bath set at 30 °C until the hexane and ethanol were evaporated away. The resulting waxy complex was then dried in a vacuum oven for 24 hours. The overall reaction is illustrated in Figure 8.

Wet iron oleate complex (the hydrate form) as obtained from the procedure described above was a reddish-brown, highly viscous liquid. It could be further purified by several methods to obtain dry iron oleate. Specifically, we purified it with ethanol, acetone, hexane, and water washes and dried in the oven at 70 °C for 24 hours. After drying, the product was a black solid. When dry iron oleate was used as a precursor, the ratio of oleic acid to iron oleate in the reaction mixture was increased as an additional stabilizing factor.

### 3.2. Synthesis of Polymorphous Iron Oxide Nanocrystals

In a standard reaction, 5 g of dry iron oleate was combined with 5.6 mL of oleic acid and 13.15 g of eicosane (boiling point 342.7 °C). The mixture was slowly heated (3 °C/min) to 50 °C under argon flow and vigorous stirring. Once the reactants had dissolved, the temperature was further increased to 342 °C at a heating rate of 3.0 °C/min. For ~20 nm polymorphous nanocrystals, the mixture was allowed to reflux for 32 min. For larger particles, the reflux time was extended, with an average growth of 2.2 nm per minute. The maximum size without adding additional reagents was 250 nm after 99 min. If the nanocrystals were allowed to cool for any amount of time before being refluxed at the same temperature again, the nanocrystal growth favored spherical morphology. After 30 min of reflux, the spheres were highly monodisperse.

### 3.3. Synthesis of Iron Oxide Nanowires

In a standard reaction, 5 g of wet iron oleate was combined with 1.6 mL of oleic acid and 13.15 g of *n-*dodecane (boiling point 216.2 °C). The mixture was slowly (3 °C/min) heated to 50 °C under argon flow and vigorous stirring. Once the reactants had dissolved, the temperature was further increased to 216 °C at a heating rate of 3 °C/min. For ~55 × 2 nm wires, the mixture was allowed to reflux for 60 min. 

### 3.4. Synthesis of Iron Oxide Spherical Nanocrystals

In a standard reaction, 5 g of wet iron oleate was combined with 1.6 mL oleic acid and 13.15 g of *n-*docosane (boiling point 370 °C). The mixture was slowly (3 °C/min) heated to 50 °C under argon flow and vigorous stirring. Once the reactants had dissolved, the temperature was further increased to 370 °C at a heating rate of 3 °C/min. For ~20 nm particles, the mixture was allowed to reflux for 32 min. For larger particles, the reflux time was extended, with an average growth rate of 1.6 nm per minute. The maximum size without adding additional reagents was 150 nm after 99 min.

### 3.5. Polyethylene Glycol (PEG) Capping of Iron Oxide Nanocrystals

PEG capping was performed following a modified procedure from [17]. The iron oxide nanocrystals were solvated in chloroform and combined with PEG using a nanocrystal to PEG mass ratio of 1:2.

## 4. Conclusions

We have synthesized and characterized PEG-capped iron oxide nanocrystals of various morphologies in paramagnetic to ferromagnetic size range that will allow further functionalization and drug conjugation. DC magnetization and ac heating power (hyperthermia characteristics) of the PEG-capped Fe_3_O_4_ nanocrystals in water have been studied. The Fe_3_O_4_ nanocrystal samples demonstrated strong ferromagnetic behavior and hysteresis losses were identified as the main mechanism of heating in hyperthermia experiments. Our hyperthermia data shows that all three nanocrystal morphologies, spheres, polymorphous nanocrystals, and wires are good candidates for thermotherapy. Significant heating was observed well within the limits for oscillating magnetic field parameters established for biological applications. The observed temperature increase for Fe_3_O_4_ nanospheres at 111.1 kHz and 25 mT after 40 min was 6 °C. If the corresponding temperature increase took place from normal human body temperature (36.6 °C) as a starting point, it would bring the local temperature up to 42.6 °C, which is right within the desirable temperature limits for the applications of medical hyperthermia (41–45 °C) [18]. Of special interest for hyperthermia applications are Fe_3_O_4_ nanowires that demonstrated a remarkable 30 °C temperature increase under magnetic field conditions that were very close to the typical values used in medical treatments. 
